# Environmental Enrichment and Physical Exercise Attenuate the Depressive-Like Effects Induced by Social Isolation Stress in Rats

**DOI:** 10.3389/fphar.2020.00804

**Published:** 2020-05-29

**Authors:** Juan C. Brenes, Jaime Fornaguera, Andrey Sequeira-Cordero

**Affiliations:** ^1^Institute for Psychological Research, University of Costa Rica, San José, Costa Rica; ^2^Neuroscience Research Center, University of Costa Rica, San José, Costa Rica; ^3^Biochemistry Department, School of Medicine, University of Costa Rica, San José, Costa Rica; ^4^Institute of Health Research, University of Costa Rica, San José, Costa Rica

**Keywords:** social isolation, environmental enrichment, physical exercise, antidepressant effect, serotonin, hippocampus, treadmill, depression

## Abstract

We assessed the antidepressant-like effects of environmental enrichment (EE) and physical exercise (PE) compared with the selective serotonin reuptake inhibitor fluoxetine against the depression-related neurobehavioral alterations induced by postweaning social isolation (SI) in rats. After 1 month of SI, rats were submitted to PE (treadmill), EE, or fluoxetine (10 mg/kg), which were compared with naïve SI and group-housed rats. After 1 month, behavior was analyzed in the open field (OFT), the sucrose preference (SPT), and the forced swimming (FST) tests. Afterward, the hippocampal serotonin contents, its metabolite, and turnover were measured. SI induced a depression-related phenotype characterized by a marginal bodyweight gain, anxiety, anhedonia, behavioral despair, and alterations of serotonin metabolism. EE produced the widest and largest antidepressive-like effect, followed by PE and fluoxetine, which were almost equivalent. The treatments, however, affected differentially the neurobehavioral domains investigated. EE exerted its largest effect on anhedonia and was the only treatment inducing anxiolytic-like effects. Fluoxetine, in contrast, produced its largest effect on serotonin metabolism, followed by its anti-behavioral despair action. PE was a middle-ground treatment with broader behavioral outcomes than fluoxetine, but ineffective to reverse the serotonergic alterations induced by SI. The most responsive test to the treatments was the FST, followed closely by the SPT. Although OFT locomotion and body weight varied considerably between groups, they were barely responsive to PE and fluoxetine. From a translational standpoint, our data suggest that exercise and recreational activities may have broader health benefits than antidepressants to overcome confinement and the consequences of chronic stress.

## Introduction

Chronic stress represents an important risk factor for many neuropsychiatric disorders, such as anxiety and affective disorders (Bao et al., 2008). Chronic social isolation (SI) from conspecifics is a highly stressful condition with multiple adverse neurobehavioral effects in humans, non-human primates, and rodents (Harlow et al., 1965; Persky et al., 1966; Morgan, 1969; Fone and Porkess, 2008). Consequently, postweaning or adolescence SI in rodents has been used to model the long-term effects of early adversity on mental health (Fone and Porkess, 2008; Mumtaz et al., 2018). SI provokes behavioral and endocrine outcomes related to anxiety- and depressive-like phenotypes (Weiss et al., 2004; Brenes et al., 2008; Lukkes et al., 2009; Quan et al., 2010). The structural and functional alterations underlying these outcomes involve several brain structures (Mumtaz et al., 2018), including the hippocampus (Quan et al., 2010; Biggio et al., 2019). Several molecular and neurochemical alterations have been observed in this brain region (for review see Mumtaz et al., 2018), including corticosterone-induced damage *via* the glucocorticoid receptor (Kamal et al., 2014), a reduction in the brain-derived neurotrophic factor (BDNF) levels (Scaccianoce et al., 2006), and the dysregulation of several neurotransmitters including glutamate (Shao et al., 2015) and serotonin (5-HT) (Fone and Porkess, 2008; Mumtaz et al., 2018). Concerning 5-HT neurotransmission, several studies showed decreased basal contents (Jaffe et al., 1993), release deficits in response to further stressor exposures (Muchimapura et al., 2002), changes in the transmitter turnover (Brenes and Fornaguera, 2009), and alterations in receptor binding or responsiveness (Wright et al., 1991; Muchimapura et al., 2003; Marsden et al., 2011). Altogether, these findings evidence a pivotal role of the 5-HTergic system in the control of SI-induced effects.

SI-associated alterations can be modified or even reversed by subsequent changes in the environment. Environmental enrichment (EE), namely, the exposure to sensory, motor, cognitive, and social stimulation higher than that received in standard housing (SH) conditions (Rosenzweig and Bennett, 1996; Simpson and Kelly, 2011), has shown to decrease locomotor hyperactivity in response to novelty (Brenes et al., 2008) and anxiety- and depression-like behaviors (Pena et al., 2006; Brenes et al., 2008; Brenes et al., 2009). EE also improves learning and memory (Leggio et al., 2005; Mora-Gallegos et al., 2015) and increases 5-HT levels in the hippocampus and the prefrontal cortex (Brenes et al., 2008; Brenes et al., 2009). Physical exercise (PE), which constitutes one of the main enriching factors of EE, is also capable of preventing anxiety- and depression-related behaviors (Greenwood et al., 2003; Fulk et al., 2004) by inducing a wide range of neurochemical effects including an increase of hippocampal 5-HT signaling (Meeusen et al., 1996; Wilson and Marsden, 1996; Béquet et al., 2001; Gomez-Merino et al., 2001; Lin and Kuo, 2013).

Although antidepressants continue to be the first-choice medication for various mental conditions (Cipriani et al., 2018), substituting or complementing psychiatric medications with non-pharmacological treatments is highly encouraged (Farah et al., 2016) because antidepressants have a moderate impact on overall health. In that regard, both animal and human studies have placed EE and PE as promising behavioral and environmental interventions in mental health capable of improving other medical conditions, including cardiovascular and metabolic diseases (Salmon, 2001; Craft and Perna, 2004; Hellemans et al., 2004; Grippo et al., 2014; Cao et al., 2017; Kühn et al., 2017; Makinodan et al., 2017; Cao et al., 2018; McDonald et al., 2018; Biggio et al., 2019). Depression is usually accompanied by a sedentary lifestyle associated with a higher risk of obesity, metabolic syndrome, and diabetes type II (Morgan, 1969; Felton et al., 2010; Luppino et al., 2010). Traditional psychiatric medication does not improve those clinical conditions, which may even worsen due to the side effects on body weight and metabolism that some antidepressants have (Craft and Perna, 2004; Mastronardi et al., 2011). In contrast, overweight and obesity increase the onset risk for depressive disorders (Luppino et al., 2010), leading to a negative feedback loop of health complications.

This study aimed to compare the antidepressant-like effects of EE and PE with the selective serotonin reuptake inhibitor fluoxetine (FLX), against the depression-related phenotype induced by chronic, postweaning SI in rats. Although a bulk of literature has already studied the antidepressant-like effects of PE or EE separately, direct comparisons of these factors have not yet been reported. Therefore, based on the calculation of sizes effects and efficacy ratios, we provided a piece of quantitative evidence about 1) the overall efficacy of EE and PE treatments relative to the classical antidepressant FLX, 2) the specific efficacy of each treatment according to the different behavioral and brain parameters assessed, and 3) the responsiveness and utility of each behavioral paradigm for screening these and other potential treatments.

## Materials and Methods

### Animals

Forty-two male Sprague-Dawley rats from LEBi Facilities (University of Costa Rica) were transported to our colony room on postnatal day (PND) 22. At PND 30, after 1-week habituation, rats were housed either single (SI, n = 33) or in groups of three (SH, n = 9) as previously reported (Brenes-Saenz et al., 2006; Brenes et al., 2008). Allocation to the groups was carried out based on body weight to avoid differences at the beginning of the experiment, considering that all treatments are capable of affecting body weight (Moraska et al., 2000; Zaias et al., 2008; Mastronardi et al., 2011; Nakhate et al., 2011). Animals were maintained in a temperature-controlled environment (20.5°C ± 1.20°C) under a 12-h light-dark cycle (lights turned on at 0600 h). Food and water were available *ad libitum* throughout the experiment. All behavioral tests were conducted and videotaped during the night cycle (19:00 to 23:00 h). Experimental procedures were done in accordance with the guidelines of the Costa Rican Ministry of Science and Technology for the Care and Use of Laboratory Animals and were approved by the Institutional Committee for Animal Care and Use of the University of Costa Rica.

### Experimental Procedure

**Figure 1:**
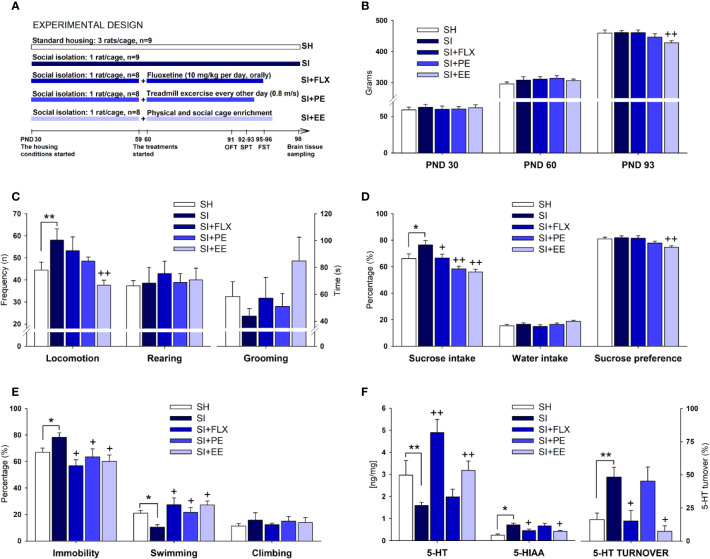
The antidepressant-like effect of EE and PE in relation to FLX. Experimental design **(A)** (see the main text for a detailed description), body weight **(B)**, the open field test **(C)**, the sucrose preference test **(D)**, the forced swimming test **(E)**, and hippocampal 5-HT parameters **(F)**. Standard housing (SH), social isolation (SI), fluoxetine (FLX), physical exercise (PE), and environmental enrichment (EE). Serotonin (5-HT), 5-hydroxyindoleacetic acid (5-HIAA), 5-HT turnover [(5-HIAA/5-HT) × 100]. Statistical differences correspond to single, planned ANOVA comparisons. SI vs. SH: *p < 0.05, **p < 0.01. SI vs. all other treatments: ^+^*p* < 0.05, ^++^*p* < 0.01.

As depicted in **Figure 1A**, both groups were kept undisturbed under their respective conditions for 30 days, comprising the entire period of the adolescence in rats (Schneider, 2013). The choice of this age range for the exposure to SI was based on the well-documented observation that chronic stress during adolescence confers susceptibility for the development of depression in adulthood (Lupien et al., 2009; Chaby et al., 2017; Sequeira-Cordero et al., 2019). At PND 60, SI animals were further divided into four groups based on body weight. We left 1 month before starting the treatments because it is a sufficiently long period for SI to induce neurobehavioral alterations to be further reversed by the treatments (for review see Fone and Porkess, 2008; Sequeira-Cordero et al., 2019), and because FLX administered during preweaning or immediately after weaning leads to disturbing and long-lasting effects on brain and behavior that we wanted to avoid (Norrholm and Ouimet, 2000; LaRoche and Morgan, 2007). The SI group remained under similar conditions (n = 9). The Fluoxetine (SI + FLX) group (n = 8) received intragastric administrations of fluoxetine hydrochloride (10 mg/kg dissolved in distilled water in a volume of 10 ml/kg; Raven, SJ, Costa Rica) during 36 consecutive days (between 09:00 and 10:00 h), whereas SI and SH groups received distilled water intragastrically at 10 ml/kg as vehicle. The FLX dose was chosen due to its effectiveness shown in previous works using the Forced Swimming Test (FST) and the Sucrose Preference Test (SPT) (Sammut et al., 2002; Cryan et al., 2005; Brenes and Fornaguera, 2009). To avoid the acute effects of the drug, animals received the last administration 14 h before the test session of the FST (see below) at PND 96. In addition, sedentary SI rats (i.e., SI rats located on a locked treadmill) were also treated with a vehicle to control the effect of the cannulation. A third group (n = 8) was subjected to treadmill exercise (SI-PE) for 30 days while maintained in SI. The exercise training started with rats being habituated to the treadmill (0.1 m/s) for three consecutive days for 45 min followed by a day off. Then, animals ran at 0.6 m/s for 50 min every other day for 14 days. Afterward, the running intensity increased to 0.8 m/s for 60 min every other day for another 14 days. To rule out possible fatigue effects, rats were not trained during the 2 days of FST (PND 95–96). On each training day, two SI-PE rats were placed on a two-lane treadmill apparatus. Simultaneously, two sedentary SI rats were placed on a locked, two-lane treadmill, which was next to the lanes of the SI runners. Finally, the EE group (n = 8) was housed in a specially designed cage (120 cm length × 70 cm width × 100 cm height) containing non-chewable plastic objects, two PVC tubes, stairs, five food dispensers, and two water bottles. Object rearrangement was carried out twice a week after the bedding change, as previously reported (Brenes-Saenz et al., 2006). The day after the FST (PND97), EE animals were placed in two SH cages (n = 4 each) until euthanasia to avoid the acute effects of EE on neurochemical results. Behavioral testing was carried out after 1 month of exposure to the treatments because it is known that a period of at least 3 to 4 weeks is necessary to obtain the expected antidepressant effects (Cao et al., 2017; Cho et al., 2017; Biggio et al., 2019; for review see Cryan et al., 2005; Fone and Porkess, 2008; Willner, 2017). Animals were subjected to the open field test (OFT), SPT, and FST from PND 91 to PND 96. The tests were organized following a stress-ascending order so that the carryover effect was the lowest. For instance, the 10-min OFT (PND 91) was considered less stressful than the 48 h of SI of the SPT (PND 92–93), and these tests were thought to be less stressful than the two sessions of the FST (PND 95–96). In terms of responsiveness, the most stressful tests are less likely to be affected by previous testing.

### Behavioral Assessment

#### OFT

At PND 91, all groups were tested for spontaneous OFT activity as described elsewhere (Brenes et al., 2008). Briefly, the testing room was dimly illuminated with one 25 W red bulb located 130 cm above the open-field floor. Each rat was placed into the center of a wood-made arena (70 × 70 × 40 cm divided into four equal squares) and allowed to explore for 10 min. The number of lines crossed with the four paws, the number of rearings (standing on hind paws), and the time spent on grooming were manually counted. The arena was cleaned with a 90% alcohol solution between tests.

#### SPT

The SPT was carried out according to Brenes and Fornaguera (2009). Rats were housed individually during 48 h (PNDs 92–93) in standard cages with one bottle containing 200 ml of 32% sucrose solution (w/v) and another one with 200 ml of tap water. Food was available *ad libitum*. After the completion of this period, sucrose (ml), water (ml), and food (g) were measured, and animals were returned to their original housing conditions. Preference was calculated as follows: Preference % = [(sucrose consumption/sucrose + water consumption) × 100]. In addition, water and sucrose consumption was expressed in percentages [(consumption/200) × 100].

#### FST

At PNDs 95–96, the FST was performed as described by Brenes et al. (2008). Animals were exposed to a 15-min pre-test session 24 h before the 5-min test. One single rat was placed into a Plexiglas cylinder (45 cm height, 31 cm diameter) filled with water (25°C ± 0.5°C) to a depth of 30 cm. After each session, rats were removed from the water, dried with a towel, and placed in a warmed chamber for 30 min before being returned to their housing cages. The water was changed after each test. The time spent on immobility (floating posture including small movements necessary to keep the animal's head above the water), swimming (the movement, usually horizontal throughout the cylinder that also includes crossing between quadrants), and climbing (vigorous upward-directed movements of the forepaws along the wall of the cylinder) were manually scored from the test session and was expressed as percent values [(seconds of each behavior/300) × 100].

### Neurochemical Analysis

The neurochemical analysis was performed as previously reported (Brenes and Fornaguera, 2008; Brenes et al., 2008). At PND 98 rats were euthanized by decapitation and brains were quickly dissected on ice, and the hippocampus was bilaterally removed. Both hemispheres were pooled, and the resulting samples were analyzed for their contents of serotonin (5-HT) and 5-hydroxyindoleacetic acid (5-HIAA) using high-performance liquid chromatography coupled with electrochemical detection (HPLC-EC). Concentrations were expressed as nanograms per milligram of wet tissue weight, whereas the 5-HT turnover was computed as follows: [(5-HIAA/5-HT) × 100].

### Statistical Analysis

Data were expressed as means ± standard error of the mean (SEM). Treatments were compared using a one-way variance analysis (ANOVA) followed by Fisher-protected, planned contrasts. The first effect of being identified was if SI differed from SH. Subsequently, we estimated the ability of each treatment to reverse the SI-induced alterations. Thus, the second comparison was between SI and FLX, PE, and EE. Eta-squared coefficients (η^2^) were obtained for each comparison to estimate the size effects per treatment, behavioral domain, and test. Subsequently, we estimated the efficacy of each treatment relative to FLX, which was expressed as ratios. The body weight was included as a covariate into an ANCOVA analysis of sucrose consumption and preference. In all statistical analyses, the significance was defined as *p* < 0.05.

## Results

### Body Weight

As groups were counterbalanced according to body weight, no significant differences were observed at the baseline level (PND 30) (**Figure 1B**). After 30 days of housing (PND 60), body weight was descriptively higher (4.5%) in SI rats than in SH counterparts, without reaching the significance level (**Figure 1B**). At PND 93, body weight continued to be descriptively higher in SI than in SH rats (4%). When comparing the treatments, FLX did not reduce body weight, with the mean values being almost the same between the two groups (**Figure 1B**). PE produced a non-significant reduction in body weight of 3.3%, whereas EE did reduce significantly this parameter in 7.2% (*F*_(1,16)_= 12.013, *p*= 0.003, η^2^ = 0.445) (**Figure 1B**). In terms of relative efficacy (**Table 1**), the effects of PE and EE on body weight were 8-and 45-folds higher than that for FLX, respectively.

**Table 1:** Effects sizes and efficacy per treatment and variable.

	Effects sizes^1^			Fold changes relative to FLX^2^	
Variables/treatments	FLX	PE	EE	PE	EE
**Body weight**	0%	8%	45%	8.0	45.0
**Locomotion**	2%	15%	44%	7.5	22.0
**Sucrose intake**	25%	58%	63%	2.3	2.5
**Sucrose preference**	0%	20%	50%	20.0	50.0
**Immobility**	50%	24%	40%	0.5	0.8
**Swimming**	41%	33%	40%	0.8	1.0
**5-HT**	69%	0%	49%	–	0.7
**5-HIAA**	25%	0%	35%	–	1.4
**5-HT turnover**	39%	0%	46%	–	1.2

^1^Effect sizes corresponded to eta-squared coefficients expressed as percentages, which derived from planned comparisons between each treatment and SI. The grey-shaded numbers indicate the coefficients that were not significant. ^2^Fold changes were estimated with the eta-squared coefficients of PE and EE in comparison to those of FLX.

### OFT

Locomotor activity was significantly higher in SI rats than in SH counterparts (*F*_(1,17)_= 4.616, *p*= 0.047, η^2^ = 0.224) (**Figure 1C**), whereas only the EE was able to restore it at the level of the SH group (*F*_(1,16)_= 11.924, *p*= 0.004, η^2^ = 0.443). No between-groups differences on rearing and grooming were detected (**Figure 1C**). As shown in **Table 1**, the effect of EE on SI-induced hyperlocomotion was 22-folds higher than that in FLX. The non-significant size effect of FLX relative to SI was only 2%, whereas for EE, it was 44%. PE produced a non-significant reduction in locomotion, which was 7.5-folds higher than that for FLX.

### SPT

SI increased significantly the sucrose intake (*F*_(1,17)_= 8.023, *p*= 0.0001, η^2^ = 0.464) as compared with SH (**Figure 1D**). All treatments were effective in reducing the SI-induced increases in sucrose consumption, with EE (*F*_(1,16)_= 25.600, *p*= 0.0001, η^2^ = 0.631) showing the largest size effect followed by PE (*F*_(1,17)_= 20.663, *p*= 0.0001, η^2^ = 0.579), and FLX (*F*_(1,16)_= 4.974, *p*= 0.041, η^2^ = 0.249). As there were differences in body weight, sucrose consumption was corrected accordingly. After doing the correction either with an index (*F*_(4,37)_= 4.153, *p*= 0.007, η^2^ = 0.310) or with an ANCOVA analysis (*F*_(4,36)_= 6.000, *p*= 0.001, η^2^ = 0.400), the significant differences among groups remained quite similar (all *p*-values< 0.05) (data not shown). SI showed no effects on water intake or sucrose preference. FLX had no effects on sucrose preference, whereas PE reduced it marginally (*p*= 0.07). Only the EE was able to reduce significantly the sucrose preference (*F*_(1,16)_= 14.780, *p*= 0.002, η^2^ = 0.496) (**Figure 1D**). After the ANOVA, such differences were still significant (*F*_(1,14)_= 7.602, *p*= 0.015, η^2^^=^ 0.352). In general, the ability of EE and PE to reverse the effects of SI on sucrose consumption was 2.5- and 2.3-folds higher than that for FLX, respectively (**Table 1**).

### FST

As shown in **Figure 1E**, SI increased immobility (*F*_(1,17)_= 5.747, *p*= 0.029, η^2^ = 0.264) and reduced swimming (*F*_(1,17)_= 14.855, *p*= 0.001, η^2^ = 0.481). All treatments were effective in reducing the immobility by increasing swimming time without affecting climbing, with FLX (*F*_(1,16)_= 14.786, *p*= 0.002, η^2^^=^ 0.496) having the largest size effect on immobility behavior followed by EE (*F*_(1,17)_= 9.900, *p*= 0.007, η^2^ = 0.398) and then by PE (*F*_(1,16)_= 4.655, *p*= 0.048, η^2^ = 0.237) (**Figure 1E**). In fact, the size effect of EE and PE were 0.8- and 0.5-fold relative to FLX, respectively (**Table 1**). No between-groups differences on climbing behavior were observed.

### Neurochemistry

**Figure 2:**
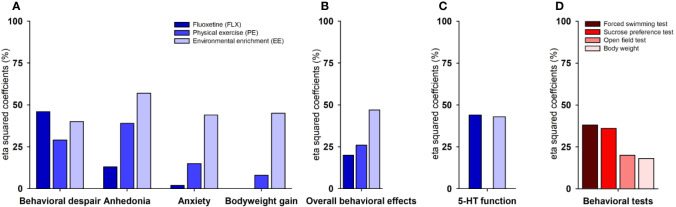
Size effects estimated per domain, test, and treatment. Size effects (i.e., eta-squared coefficients) refer to the proportion of variance explained by the treatments. Each behavioral domain comprised the average size effects of all variables that differed significantly between groups **(A)**. The overall average of size effects for all behavioral domains **(B)**. Hippocampal serotonin (5-HT) function comprising the average size effects of 5-HT, 5-hydroxyindoleacetic acid (5-HIAA), and 5-HT turnover [(5-HIAA/5-HT) × 100] **(C)**. The average of size effects per behavioral test or parameter **(D) (F)**. Fluoxetine (FLX), physical exercise (PE), and environmental enrichment (EE).

SI reduced the hippocampal concentration of 5-HT (*F*_(1,17)_= 7.265, *p*= 0.0001, η^2^ = 0.440), and increased 5-HIAA levels (*F*_(1,17)_= 6.208, *p*= 0.001, η^2^ = 0.402) and 5-HT turnover (*F*_(1,17)_= 5.455, *p*= 0.001, η^2^ = 0.371) (**Figure 1F**). FLX and EE were able to reverse the neurochemical changes induced by SI, with FLX producing the largest effect on 5-HT concentration (*F*_(1,16)_= 33.282, *p*= 0.0001, η^2^ = 0.689) and EE on 5-HIAA (*F*_(1,16)_= 8.144, *p*= 0.012, η^2^ = 0.352) and 5-HT turnover (*F*_(1,16)_= 12.962, *p*= 0.003, η^2^ = 0.464) (**Figure 1F**). On the contrary, PE was not capable of remediating the SI-induced neurochemical changes. In terms of relative efficacy (**Table 1**), FLX exerted the largest effect on restoring 5-HT concentration, followed only by EE (0.7-fold relative to FLX). In contrast, EE had an effect 1.4- and 1.2-folds higher than FLX on normalizing the alterations in the 5-HIAA and 5-HT turnover, respectively. The comparison of the three 5-HT parameters revealed that the 5-HT concentration is the most responsive variable to the treatments (39%), followed by the turnover (28%), and the metabolite (20%) (**Table 1** and **Figure 2C**).

### Relative Efficacy per Treatment and Parameter

To compare the different behavioral domains measured, we averaged the size effects of the behavioral parameters belonging to the same test, when appropriate (**Figure 2**). On anhedonia (**Figure 2A**, left panel), EE produced the largest size effect of all treatments (57%), followed by PE (39%) and FLX (13%). On behavioral despair (**Figure 2A**, middle left panel), FLX exerted the largest effect (46%), followed by EE (40%) and PE (29%). On anxiety (44%) and body weight (45%) (**Figure 2A**, middle right and right panels), only EE produced significant effects, whereas PE affected those parameters marginally (15% and 8%, respectively). FLX, in contrast, was completely ineffective in reverting the SI-induced changes in locomotion and body weight (0–2%). Then, to summarize the overall antidepressant-like action, we averaged the size effects of all behavioral domains (**Figure 2B**). As expected, EE appeared as the most effective treatment (47%) followed by PE (26%), and FLX (20%). The efficacy relative to FLX was 2.3- and 1.3-folds higher for EE and PE, respectively. To determine the most responsive test, we compared the behavioral domains among each other, irrespective of the treatments (**Figure 2D**). The FST was the most responsive test with an average size effect of 38%, followed by the SPT with 36%, the OFT with 20%, and the body weight with 18%. When averaging the size effects of the three 5-HT parameters, we found that FLX (44%) and EE (43%) exerted almost identical effects with a comparable efficacy (i.e., 0.98-fold relative to FLX) (**Table 1** and **Figure 2C**).

## Discussion

Here, we investigated the ability of two non-pharmacological treatments to reverse the depressive-like phenotype induced by chronic SI in rats. As a positive control, the antidepressant FLX was used to compare the treatments' efficacy. For body weight, no significant differences between SI and SH rats were observed, in agreement with other reports in rats and mice (Sahakian et al., 1982; Pérez et al., 1997; Hellemans et al., 2004; Nonogaki et al., 2007; Schiavone et al., 2016). Nevertheless, other studies have found a significant increase in body weight in SI rats (Fiala et al., 1977; Nakhate et al., 2011). In the current experiment, our SI rats maintained a consistent, yet non-significant increase of ~4% in body weight compared to SH animals (**Figure 1B**). Such an increase closely resembles the diagnostic criterion for major depression (i.e., a change of more than 5% in body weight in a month, APA, 2013); however, the physiological relevance of a 4% change in body weight in rats remains undetermined and should be addressed experimentally. Regarding the antidepressant treatments, only EE rats weighed significantly less than the SI group after our protocol. This EE-induced reduction in body weight gain has been observed previously (Zaias et al., 2008; Zakharova et al., 2012; Harati et al., 2013), and could be explained by a higher physical activity, which is a pivotal enriching factor (Di Garbo et al., 2011; Crofton et al., 2015), as discussed elsewhere in this Research Topic (Rojas-Carvajal et al., 2020). An alternative explanation has been proposed in which EE induces a crowding-related, partial food-deprivation effect (Brown and Grunberg, 1996). However, such a distress-based explanation would imply the presence of a hardship state in the EE rats, which opposes the behavioral phenotype reported here. PE alone failed to reduce the bodyweight gain significantly, agreeing with several studies (Hansalik et al., 2006; Salim et al., 2010; Lalanza et al., 2015) but not with others (Brown et al., 2007; Moraska et al., 2000; Chennaoui et al., 2002). In fact, the marginal effect of PE was half as large as the one produced by EE and could be explained by differences in the experimental design regarding the intensity and duration of PE, as discussed elsewhere (Lalanza et al., 2015). Although our treadmill protocol could be considered intense (36 m/min from days 1 to 14, and 48 m/min from days 15 to 28), the exposures were carried out every other day, which could have impeded a reduction in body weight contrary to the daily EE exposures. Although further research is needed, our findings suggest that moderate but sustained physical activity could reduce body weight more efficiently than intense but short sessions of exercise interspersed over days.

Animals housed in SI showed an emotional and behavioral dysregulation evidenced by increased locomotion in the OFT, sucrose consumption in the SPT, and immobility in the FST, supporting the well-known anxiogenic and depressogenic effects of chronic SI and in agreement with a huge body of findings (Sahakian et al., 1977; Yates et al., 1991; Hall et al., 1997; Hall et al., 1998; Heidbreder et al., 2000; Silva-Gómez et al., 2003; Brenes-Saenz et al., 2006; Brenes and Fornaguera, 2008; Lukkes et al., 2009; Kokare et al., 2010; Takatsu-Coleman et al., 2013). As a model of depression, SI is quite reliable and easy to implement as compared with the chronic unpredictable stress (CUS) or the social defeat (SD) models, which have laborious protocols with many versions available, sometimes leading to inconsistent results. In fact, SI represents the most replicated stressor within the CUS model and might account for several of its results (for a discussion, see Sequeira-Cordero et al., 2019). In the SD model, SI after the defeating experience is essential for maintaining the physiological and behavioral sequels of social stress (de Jong et al., 2005). Thus, our findings demonstrate that SI is capable of inducing a depression-like phenotype even in the absence of other stressors.

OFT hyperlocomotion is interpreted as an augmented response to mild stress, which may result from an impairment in the arousal-inhibition system and novelty habituation (Rojas-Carvajal et al., 2018; Sequeira-Cordero et al., 2019). Among all treatments, only EE was able to produce an anxiolytic-like effect by attenuating the SI-induced hyperlocomotion, in agreement with previous reports (Brenes-Saenz et al., 2006; Brenes et al., 2008; Brenes and Fornaguera, 2009). In second place was PE, which reduced locomotion descriptively, with an effect 13% higher than that for FLX. Although such an effect did not yield the significance level, it suggests that a more frequent PE protocol would have led to significant differences in locomotion as it has been reported by others (Hoffmann et al., 1987; Duman et al., 2008). FLX exerted no effects on SI-induced hyperlocomotion in agreement with the evidence from SI (Brenes and Fornaguera, 2009) and other models of depression (West and Weiss, 1998; Mar et al., 2002; Ghorpade et al., 2011; Baek et al., 2015). In unstressed subjects, conversely, subchronic and chronic FLX treatment has been found to reduce locomotion (Dulawa et al., 2004; Brookshire and Jones, 2009; Sass and Wörtwein, 2012), suggesting that stress-induced alterations in exploratory and locomotor activity are less responsive to FLX. However, negative results have also been obtained in unstressed rodents (Homberg et al., 2011; Allen et al., 2012; Santos et al., 2012; Gray and Hughes, 2015) highlighting the relevance of methodological factors (e.g., species or strains, sex, age, doses, duration, administration route, and behavioral protocols) in order to replicate these findings.

When SI rats were challenged with an inescapable and more severe stressor (e.g., FST), a passive-coping response was rather displayed (i.e., immobility), which is interpreted as a depressogenic effect (Porsolt et al., 1978). Both EE and PE exerted an effective anti-behavioral despair action (i.e., FST), which was smaller than that of FLX (**Table 1**). These findings are in agreement with the evidence about the anti-behavioral despair effects of EE and PE (Porsolt et al., 1978; Brenes-Saenz et al., 2006; Brenes and Fornaguera, 2008; Duman et al., 2008; Brenes and Fornaguera, 2009; Cunha et al., 2013; Lee et al., 2015) and with the expected outcome of FLX on this test (for a review see Cryan et al., 2005). Our results suggest that the FST is still an effective test for behavioral screening despite the critics received (Reardon, 2019), and poses a challenge for implementing better behavioral paradigms.

In the SPT, EE and PE reversed the SI-induced increases in sucrose consumption to a greater extent than FLX. The effect of SI in this paradigm can be considered the expected outcome in rats (Hall et al., 1997; Hall et al., 1998; Van den Berg et al., 2000; Brenes and Fornaguera, 2009; Sequeira-Cordero et al., 2019). The increased sucrose intake may reflect a dysregulation in the reward threshold produced by SI. Accordingly, consumption could have increased to compensate for the inability to experience the same rewarding sensations that non-SI animals would experience, and therefore, it can be interpreted as an index of anhedonia. In that regard, we have found that SI rats drinking the highest amount of sucrose had the lowest dopamine contents in the ventral striatum, one of the main reward centers of the brain (Brenes and Fornaguera, 2008). CUS –a model in which SI rats are randomly exposed different stressors– has been shown to increase the intracerebral stimulation threshold of the ventral tegmental area (Moreau et al., 1992), the primary source of dopaminergic cell bodies projecting to the ventral striatum. These findings suggest that chronic stress based on SI reduces dopamine available for reward signaling. As sucrose intake also correlates positively with FST immobility (Brenes and Fornaguera, 2008), and FLX is able to reverse the SI-induced increases in sucrose consumption and immobility (current results and Brenes and Fornaguera, 2009), we take these effects as indicative of a depressive-like phenotype, which was reversed by EE and PE. We are aware that the reduction in sucrose intake or preference is frequently observed in CUS (Katz, 1982; Willner, 2017). In our hands, however, we have obtained almost the same results (e.g., increase in sucrose intake) after SI or CUS, using a sucrose solution at 1% or 32% or employing Wistar or Sprague-Dawley rats (Brenes and Fornaguera, 2008; Brenes and Fornaguera, 2009; Sequeira-Cordero et al., 2019). In current and previous experiments (Brenes and Fornaguera, 2009; Sequeira-Cordero et al., 2019), sucrose preference was less responsive to the treatments. The difference between sucrose intake and preference may result from the fact that even severely stressed animals still prefer sucrose over water (Katz, 1982; Willner et al., 1987; for review see Willner, 2017), which turns preference into a less informative parameter than consumption. Indeed, many CUS studies –even from the same laboratory– have reported either consumption or preference but not both, and sometimes with inconsistent results (Willner et al., 1987; Muscat and Willner, 1989; Papp et al., 1991; D'Aquila et al., 1994; for review see Willner, 2017). Although in the current experiment the SPT (i.e., sucrose intake) was one of the two most responsive tests (together with the FST), it has the disadvantage of being quite inconsistent within and between laboratories.

At the neurochemical level (**Figure 1F**), SI reduced the hippocampal 5-HT contents and increased its metabolite (5-HIAA) and turnover, in agreement with previous reports (Muchimapura et al., 2002; Brenes and Fornaguera, 2009; Brenes et al., 2009). FLX restored the SI-induced reduction in 5-HT concentration to the largest extent, followed by EE, which had a minor yet significant effect on this parameter. For the 5-HIAA concentration and 5-HT turnover, EE outperformed FLX on normalizing these alterations. When averaging the size effects of the three 5-HT parameters, FLX and EE showed an equivalent efficacy (**Table 1** and **Figure 2C**). Out of the three serotonergic parameters, the concentration of 5-HT appeared as the most responsive variable to the treatments followed by the turnover and the metabolite, suggesting that measuring only the 5-HT contents might be enough for detecting the effects of stress (e.g., SI) and the treatments. Our SI results support the well-accepted view that alterations in the 5-HT function (e.g., 5-HT levels, receptors or its transporter)—in the hippocampus and other brain regions—play a key role in the development of depression and depression-like behaviors (Albert et al., 2012; Dean and Keshavan, 2017). As expected, FLX produced the largest increase in extracellular concentrations of 5-HT, with its concomitant behavioral outcomes being particularly noticeable on immobility and swimming behaviors (Cryan et al., 2005). The broader and stronger behavioral effects of EE may result from its action on both 5-HT and non–5-HT neurotransmission, as reported elsewhere (for review see Simpson and Kelly, 2011). Although similar effects of EE on 5-HT, 5-HIAA, or its turnover have been described (Galani et al., 2007; Brenes et al., 2008; Brenes et al., 2009; Chourbaji et al., 2012; Leger et al., 2014), many discordant results are showing either a lack of effects (Chourbaji et al., 2012; Leger et al., 2014) or a reduction in the 5-HT and 5-HIAA levels (Galani et al., 2007). These discrepancies can be due to methodological aspects related to the EE (e.g., cage size, housing density, protocol duration, age, and species) or with the methods for tissue sampling and neurochemical analysis (Simpson and Kelly, 2011).

On the other hand, PE produced no changes in 5-HT parameters, which contrasts with its antidepressant-like action at the behavioral level. Such effects may be due to the involvement of other ligands, such as the BDNF and the endocannabinoids, which are potentiated by PE (Fang et al., 2013; Ferreira-Vieira et al., 2014). Although PE increases 5-HT neurotransmission, the concentrations used to return to baseline within 2 h after the treadmill exposure (Meeusen et al., 1996; Wilson and Marsden, 1996; Béquet et al., 2001; Gomez-Merino et al., 2001), suggesting that the alterations are transient and intensity- or duration-dependent (Lee et al., 2013; Otsuka et al., 2016). Thus, to measure only the cumulative effects of chronic PE, we let 2 days between the last treadmill exposure and euthanasia, finding no changes in 5-HT and its metabolite. It must be emphasized that the vast majority of the neurochemical effects of PE in the literature have been obtained in non-stressed subjects. As we did not include a group of PE rats housed in groups, our results are not entirely comparable with most of the PE studies. Our data show, at least, that our PE protocol was not enough to reverse the neurochemical alterations induced by SI. It can be argued, alternatively, that behavioral testing disguised the neurochemical effects of PE, as behavioral paradigms on its own can alter the 5-HT transmission (Smolders et al., 2001; Yoshitake et al., 2004; Bouwknecht et al., 2007). However, such an explanation could be less plausible, as our animals remained undisturbed for 2 days until euthanasia, and the monoaminergic changes induced by the behavioral testing are not supposed to last as long as to be detected 48 h later (Joëls and Baram, 2009).

We are aware of some caveats reducing the extent of our results. First, the treatments were not completely comparable as FLX and EE were administered daily, whereas the treadmill sessions took place every other day. This difference could have reduced the actual effects of PE, making the comparison with EE biased. We spaced out the PE sessions to avoid the distress associated with forced exercise; however, we cannot rule out that such an effect has already occurred counteracting somehow the benefits of PE. Second, the lack of additional groups impeded a sharper dissection of the neurobehavioral effects induced by each treatment. For instance, we studied the combined effect of social and physical EE instead of using two separate groups for each factor (e.g., SI physically enriched and SH housed in larger groups). Third, we restricted our brain analysis to ex-vivo 5-HT measurements and only in the hippocampus, which had undoubtedly limited the identification of new cellular and molecular mechanisms of EE and PE and potential targets for pharmacological screening. These limitations should be considered in future research for a better understanding of the antidepressant-like action of EE and PE.

In conclusion, we demonstrated that chronic SI induced a depression-related phenotype characterized by a marginal increase in body weight, anxiety, anhedonia, behavioral despair, and alterations of hippocampal 5-HT metabolism. EE produced the widest and largest antidepressive-like effect, followed by PE and FLX, which were almost equivalent. The treatments, however, affected differentially the neurobehavioral domains investigated. EE exerted its largest effect on anhedonia and was the only treatment inducing anxiolytic-like effects. FLX, in contrast, produced its largest effect on hippocampal 5-HT parameters, followed by its anti-behavioral despair action. PE was a middle-ground behavioral treatment that was behind EE in all parameters and outperformed FLX on anhedonia. Surprisingly, at the neurochemical level, PE was unable to reverse the alterations induced by SI. The most responsive test to assess the effects of the treatments was the FST, followed closely by the SPT. In third and fourth place appeared two parameters with almost identical size effects: the OFT locomotion and the body weight. Although these parameters varied significantly among treatments, they were less responsive to PE and almost unresponsive to the FLX, which questions their utility for screening new drugs with potential antidepressant action based on FLX efficacy. Finally, by estimating the size effects and the ratios for relative efficacy we were able to provide a piece of quantitative evidence about 1) the overall efficacy of each treatment, 2) the specific efficacy according to the different behavioral domains assessed, and 3) the responsiveness and utility of each behavioral paradigm for screening these and other potential treatments. From a translational standpoint, our data suggest that including exercise, and social and non-social recreational activities may have a broader range of health benefits than antidepressants, highlighting the relevance of substituting or complementing traditional medications with non-pharmacological treatments.

## Data Availability Statement

The datasets generated for this study are available on request to the corresponding author.

## Ethics Statement

The animal study was reviewed and approved by Institutional Committee for Animal Care and Use of the University of Costa Rica.

## Author Contributions

JB conceived, designed, and conducted the experiments. JB and AS-C analyzed the raw behavioral data. JF performed the neurochemical analysis. All authors wrote, reviewed, and approved the manuscript.

## Conflict of Interest

The authors declare that the research was conducted in the absence of any commercial or financial relationships that could be construed as a potential conflict of interest.
